# Potent anti-tumor effects of a dual specific oncolytic adenovirus expressing apoptin in vitro and in vivo

**DOI:** 10.1186/1476-4598-9-10

**Published:** 2010-01-20

**Authors:** Li Xiao, Liu Yan, Wen Zhongmei, Li Chang, Lu Huijun, Tian Mingyao, Jin Kuoshi, Sun Lili, Gao Pegn, Yang Encheng, Xu Xiaohong, Kan Shifu, Wang Zhuoyue, Wang Yuhang, Jin Ningyi

**Affiliations:** 1 Genetic Engineering Laboratory of PLA, Academy of Military Medical Sciences of PLA, Changchun, China; 2 Department of Gastroenterology, the First Hospital of Jilin University, Changchun, China; 3 Department of Respiratory Medicine, the First Hospital of Jilin University, Changchun, China; 4 Head and Neck Surgery, The Tumor hospital of Jilin province, Changchun, China; 5 Department of Hematology and Oncology, People's Hospital of Jilin Province, Changchun, China; 6 The Hospital of Digestive Diseases, Hospital of Heilongjiang Province, Harbin, China

## Abstract

**Background:**

Oncolytic virotherapy is an attractive drug platform of cancer gene therapy, but efficacy and specificity are important prerequisites for success of such strategies. Previous studies determined that Apoptin is a p53 independent, bcl-2 insensitive apoptotic protein with the ability to specifically induce apoptosis in tumor cells. Here, we generated a conditional replication-competent adenovirus (CRCA), designated Ad-hTERT-E1a-Apoptin, and investigated the effectiveness of the CRCA a gene therapy agent for further clinical trials.

**Results:**

The observation that infection with Ad-hTERT-E1a-Apoptin significantly inhibited growth of the melanoma cells, protecting normal human epidermal melanocytes from growth inhibition confirmed cancer cell selective adenoviral replication, growth inhibition, and apoptosis induction of this therapeutic approach. The *in vivo *assays performed by using C57BL/6 mice containing established primary or metastatic tumors expanded the *in vitro *studies. When treated with Ad-hTERT-E1a-Apoptin, the subcutaneous primary tumor volume reduction was not only observed in intratumoral injection group but in systemic delivery mice. In the lung metastasis model, Ad-hTERT-E1a-Apoptin effectively suppressed pulmonary metastatic lesions. Furthermore, treatment of primary and metastatic models with Ad-hTERT-E1a-Apoptin increased mice survival.

**Conclusions:**

These data further reinforce the previously research showing that an adenovirus expressing Apoptin is more effective and advocate the potential applications of Ad-hTERT-E1a-Apoptin in the treatment of neoplastic diseases in future clinical trials.

## Background

Despite the many advances achieved in cancer prevention, much improvement is desired for present treatment protocols, including surgery, chemotherapy, and radiotherapy. These procedures may be effective in the early stages of disease, but are always palliative and fall short of eradicating the various malignant subpopulations in neoplastic conditions [1]. The current realities have highlighted the need for more novel cancer therapies to induce effective responses in clinical trials. Gene therapy is a promising strategy for patients resistant to traditional therapies because they target defects in malignant cells, selectively correcting or eradicating defective tissues [2]. However, efficacy and specificity remain major challenges for existing cancer gene therapy [3]. Oncolytic virotherapy is an attractive drug platform of cancer gene therapy consistent with the both goals [4,5]. The oncolytic viruses exhibit an ability to replicate selectively in tumor tissues to the exclusion of normal cells [5]. Furthermore, they can be genetically manipulated to express multiple cancer cell-specific therapeutic elements [6]. These characteristics demonstrate the utility of oncolytic virotherapy in the clinics and provide the basis for novel approaches to cancer gene therapy.

Adenovirus-based vectors are the most widely used platforms in gene delivery [5]. However, non-replicating adenoviruses are seldom effective in eradicating tumor cells [7]. Therefore, very high concentrations and multiple administrations are generally needed to produce significant anti-tumor responses; such regimens, however, often induce anti-viral immune responses that result in the neutralization of the viral vectors in subsequent immunizations and toxicity to the tissues [8,9]. To circumvent these limitations, conditional replication-competent adenoviruses (CRCA) have been developed and are being extensively evaluated; these viruses replicate specifically in tumor cells with subsequent oncolysis and release of virus progeny to further infect and destroy neighboring cancer cells [7,10]. Furthermore, it has been recently suggested that antibodies which neutralize replication-incompetent adenoviruses have limited effects on the replication-competent adenoviruses [7,11]. It is therefore reasonable to anticipate that replication-selective tumor-specific adenoviruses would have potent effects in cancer gene therapy.

In this study, we constructed an oncolytic adenovirus using a cancer-specific promoter (human telomerase reverse transcriptase promoter) and a cancer cell selective apoptosis-inducing gene (Apoptin) that demonstrated significant anti-tumor activity toward solid tumors and metastatic nodules. Telomerase activity is strikingly higher in about 90% of cancers than in normal cells, and it has been widely used as a tumor marker. One of the three subunits of telomerase, human telomerase reverse transcriptase (hTERT), is the determinant of the telomerase activity and is highly active in immortalized cell lines and over 85% of human cancers. Therefore, its promoter has been used for tumor specific expression of transgenes. Apoptin, the product of the chicken anemia virus VP3 gene, shows specificity and efficiency toward a wide range of transformed and malignant cells of human origin, including hepatomas, lymphomas, cholangiocarcinomas, melanomas, and breast, lung, and colon carcinomas, while sparing non-transformed primary cells such as fibroblasts, keratinocytes, or smooth muscle cells [12-14]. Preliminary studies have demonstrated the effect of Apoptin inserted in various vectors on restricting manifold tumors, which make it attractive for cancer gene therapy [15,16].

In this study, we focused on melanoma, which has doubled over the past decades and the incidence is increasing more rapidly than any other cancer [17]. Although melanoma skin cancer can be cured by surgical excision and efforts have been carried out to develop new therapies, the prognosis of patients are poor and the 5-year survival rates range from merely 10% to 50% [17,18]. Moreover, the patients with regional lymphatic or metastatic disease respond poorly to conventional radiation and chemotherapy [18]. Here, we describe the construction of a CRCA, hereafter referred to as Ad-hTERT-E1a-Apoptin, that can target tumors systemically and selectively induce apoptosis both *in vitro *and *in vivo*. We showed that infection of melanoma cells with Ad-hTERT-E1a-Apoptin resulted in a significant induction of apoptosis and induced growth suppression of melanoma cells. To determine whether Ad-hTERT-E1a-Apoptin can cause tumor regression, we also performed multiple intratumoral injections or systemic administrations of Ad-hTERT-E1a-Apoptin in subcutaneous homoplastic graft or lung metastases models, respectively. Our findings indicate that Ad-hTERT-E1a-Apoptin represents a potentially applicable anti-cancer agent for the treatment of primary and metastatic melanoma and may be of clinical value toward other neoplastic diseases.

## Results

### Generation of the recombinant adenoviruses

**Figure 1:**
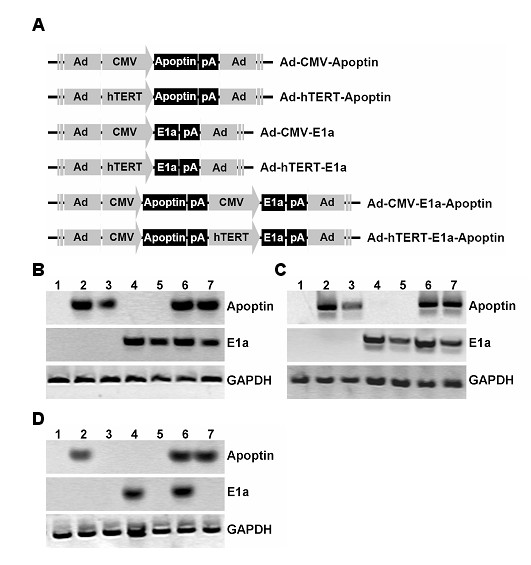
**Schematic of the recombinant adenoviruses and the adenovirus-mediated transgene expression**. (A) Schematic diagram depicting the organization elements in the recombinant adenoviruses. Polyadenylation signal sequence is designated as pA. The indicated cells were infected with Ad-mock (control) or indicated recombinant viruses at a MOI of 100 for 48 h. Western blot analysis was performed to detect (B) Apoptin or E1a protein from A375 cells, (C) Apoptin or E1a expression in B16 cells, or (D) Apoptin or E1a expression in HEM cells. Infection of normal HEM human epidermal melanocytes (D) with Ad-CMV-E1a or Ad-CMV-E1a-Apoptin, but not Ad-hTERT-E1a or Ad-hTERT-E1a-Apoptin, resulted in production of E1a proteins, whereas in A375 (B) and B16 (C) melanoma cells, infection with all these replication-competent recombinant adenoviruses generated E1a proteins. In HEM cells (D), infection with Ad-CMV-E1a-Apoptin and Ad-CMV-Apoptin resulted in Apoptin production, whereas infection with Ad-hTERT-Apoptin or Ad-hTERT-E1a-Apoptin resulted in barely detectable of Apoptin production. In A375 (B) and B16 (C) cells, infection with Ad-CMV-Apoptin, Ad-hTERT-Apoptin, Ad-CMV-E1a-Apoptin, or Ad-hTERT-E1a-Apoptin generated significant Apoptin production. 1. Ad-mock; 2. Ad-CMV-Apoptin; 3. Ad-hTERT-Apoptin; 4. Ad-CMV-E1a; 5. Ad-hTERT-E1a; 6. Ad-CMV-E1a-Apoptin; 7. Ad-hTERT-E1a-Apoptin.

The structures of the recombinant adenoviruses constructed for this study are presented in Figure 1A, including Ad-hTERT-E1a-Apoptin and Ad-CMV-E1a-Apoptin, in which the E1a gene is under the control of the hTERT promoter or CMV promoter, respectively, and Apoptin is controlled by a CMV promoter. We generated two additional control replicating adenoviruses without Apoptin, Ad-hTERT-E1a and Ad-CMV-E1a, in which E1a is expressed by the hTERT or CMV promoters, respectively. The two replication-incompetent adenoviruses (lacking the E1a gene) were Ad-hTERT-Apoptin and Ad-CMV-Apoptin, in which hTERT promoter or CMV promoter drove Apoptin expression, respectively. An empty adenovirus vector (designated Ad-mock) was used as control.

### Transgene expression and characterization of recombinant adenoviruses

In the melanoma cell lines (A375 and B16), infection with Ad-hTERT-E1a-Apoptin, Ad-CMV-E1a-Apoptin, Ad-hTERT-Apoptin and Ad-CMV-Apoptin resulted in significant production of Apoptin proteins (15 kDa), whereas no Apoptin protein was detected after Ad-hTERT-E1a, Ad-CMV-E1a or Ad-mock infection (Figure 1B and 1C). In the HEM cell line, infection with Ad-CMV-Apoptin, Ad-hTERT-E1a-Apoptin and Ad-CMV-E1a-Apoptin, but not Ad-hTERT-Apoptin, Ad-hTERT-E1a, Ad-CMV-E1a or Ad-mock, produced Apoptin protein (Figure 1D). A375 or B16 cells infected with Ad-hTERT-E1a-Apoptin, Ad-CMV-E1a-Apoptin, Ad-hTERT-E1a and Ad-CMV-E1a produced E1a proteins (36 kDa); however, cells infected with Ad-hTERT-Apoptin, Ad-CMV-Apoptin or Ad-mock did not (Figure 1B and 1C). Infection of HEM with Ad-CMV-E1a-Apoptin or Ad-CMV-E1a resulted in E1a production, whereas infection with Ad-hTERT-E1a-Apoptin, Ad-hTERT-E1a, Ad-hTERT-Apoptin, Ad-CMV-Apoptin or Ad-mock resulted in barely detectable E1a expression (Figure 1D). These results demonstrated that Ad-hTERT-E1a-Apoptin had cancer cell-selective replication properties, and the Apoptin transgene was effectively expressed.

### The cancer-specific inhibition activity of Ad-hTERT-E1a-Apoptin

**Figure 2:**
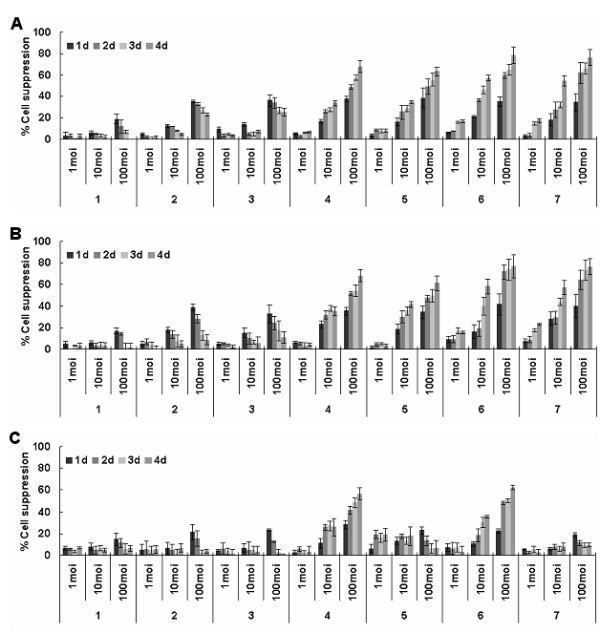
**Assessment of the selective inhibition effect of Ad-hTERT-E1a-Apoptin on melanoma cells**. Effects of the different MOIs and infection times on (A), A375 cell viability, (B) B16 cell viability, and (C), on HEM cell viability. Cells were seeded in 96-well plates (1 × 10^4 ^cells/well) one day before cells were infected with various concentrations (1 MOI, 10 MOI, and 100 MOI) of the indicated adenoviruses. Tumor viability was measured every day over a 4 days period by MTT colorimetric assay and all measurements were performed in triplicate. Data are presented as mean ± SD. In normal HEM human epidermal melanocytes (C), infection with Ad-CMV-E1a or Ad-CMV-E1a-Apoptin, but not Ad-CMV-Apoptin, Ad-hTERT-Apoptin, Ad-hTERT-E1a, or Ad-hTERT-E1a-Apoptin, induced growth inhibition. In contrast, in A375 (A) and B16 (B) melanoma cells, Ad-hTERT-E1a-Apoptin, Ad-hTERT-E1a-Apoptin, Ad-CMV-E1a, and Ad-hTERT-E1a infection resulted in significant growth inhibition. 1. Ad-mock; 2. Ad-CMV-Apoptin; 3. Ad-hTERT-Apoptin; 4. Ad-CMV-E1a; 5. Ad-hTERT-E1a; 6. Ad-CMV-E1a-Apoptin; 7. Ad-hTERT-E1a-Apoptin.

As expected, with longer infection times, growth of A375 or B16 cells infected with Ad-CMV-E1a, Ad-hTERT-E1a, Ad-CMV-E1a-Apoptin and Ad-hTERT-E1a-Apoptin were inhibited. However, cells infected with replication-incompetent adenoviruses (Ad-CMV-Apoptin, Ad-hTERT-Apoptin and Ad-mock) gradually resumed growth after 2 d. In contrast, the growth of HEM cells infected only with Ad-CMV-E1a or Ad-CMV-E1a-Apoptin, in which viral replication is controlled by the CMV promoter, were suppressed. As shown in Figure 2, the cell viabilities were dependent on the MOI of the recombinant adenoviruses to some extent. In A375 and B16 cells, infection with Ad-CMV-E1a or Ad-hTERT-E1a at an MOI of 10 or 100 induced cell growth inhibition of 30%-40% and 60%-70% after 4 d, respectively. When infected with 1 MOI or 10 MOI of Ad-CMV-E1a-Apoptin or Ad-hTERT-E1a-Apoptin, cell growth was inhibited by 10%-20% and 50%-60% after 4 d, respectively, and the growth of cells infected with 100 MOI was almost blocked (70%-80%). However, in HEM cells, the dose-dependent inhibition was only observed in the Ad-CMV-E1a and Ad-CMV-E1a-Apoptin experimental groups. It is notable that, in A375 and B16 cells, the replication-competent adenoviruses were much more potent than the replication-incompetent adenoviruses in cancer cell suppression. Furthermore, the growth inhibition of 10 MOI Ad-CMV-E1a-Apoptin or Ad-hTERT-E1a-Apoptin infected cells were similar to that of 100 MOI ofAd-CMV-E1a or Ad-hTERT-E1a, indicating that the adenoviruses with the dual cancer-specific genes were more effective than the normal replication-incompetent adenoviruses in inhibiting cell growth. That is, Ad-hTERT-E1a-Apoptin could replicate specifically in melanoma cells (A375 and B16) and restrict the cell growth selectively, and exhibited higher tumor-specific killing activity than the control viruses. The interaction of infection time and MOI was complex and synergistic, and cell viability showed an apparent dependent relationship on both factors.

### Effect of Ad-hTERT-E1a-Apoptin on apoptosis of melanoma cells

**Figure 3:**
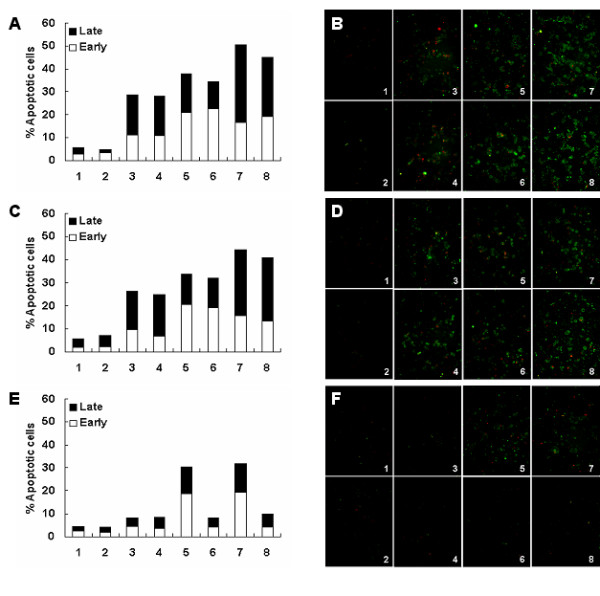
**Induction of apoptosis selectively in melanoma cells by Ad-hTERT-E1a-Apoptin**. (A) Flow cytometry analysis of A375 cells infected with the recombinant adenoviruses. (B) Fluorescence images of the adenovirus-infected A375 cells stained with Annexin V/PI. (C) Flow cytometry analysis of B16 cells infected with recombinant adenoviruses. (D) Fluorescence images of the adenovirus-infected B16 cells stained with Annexin V/PI. (E) Flow cytometry analysis of HEM cells infected with recombinant adenoviruses. (F) Fluorescence images of the adenovirus-infected HEM cells stained with Annexin V/PI. Representative images of three independent experiments at 100× magnification were used to show Annexin V binding. Infection with only Ad-CMV-E1a and Ad-CMV-E1A-Apoptin elevated the percentage of apoptotic normal HEM human epidermal melanocytes (E and F). However, all of the recombinant adenoviruses, except for Ad-mock, resulted in significant apoptosis in A375 (A and B) and B16 (C and D) melanoma cells. 1. Control; 2. Ad-mock; 3. Ad-CMV-Apoptin; 4. Ad-hTERT-Apoptin; 5. Ad-CMV-E1a; 6. Ad-hTERT-E1a; 7. Ad-CMV-E1a-Apoptin; 8. Ad-hTERT-E1a-Apoptin.

As shown in Figure 3, infection of all recombinant adenoviruses resulted in apoptosis of A375 and B16 cells, whereas in HEM cells, only infection with Ad-CMV-E1a and Ad-CMV-E1a-Apoptin induced apoptosis. In A375 and B16 cells, infection with the adenoviruses expressing Apoptin were observed predominantly in a late apoptotic stage; however, infection with Ad-CMV-E1a or Ad-hTERT-E1a were seen in mainly an early apoptotic stage. In HEM cells, because of the lost of the apoptotic inducing effect of Apoptin, Ad-CMV-E1a-Apoptin infected cells presented mainly in an early apoptotic stage similar to the Ad-CMV-E1a infected cells. These results indicated that Ad-hTERT-E1a-Apoptin induced apoptosis in melanoma cells specifically and induced apoptosis more rapidly than the control viruses.

### Ad-hTERT-E1a-Apoptin suppressed subcutaneous primary tumor growth

**Figure 4:**
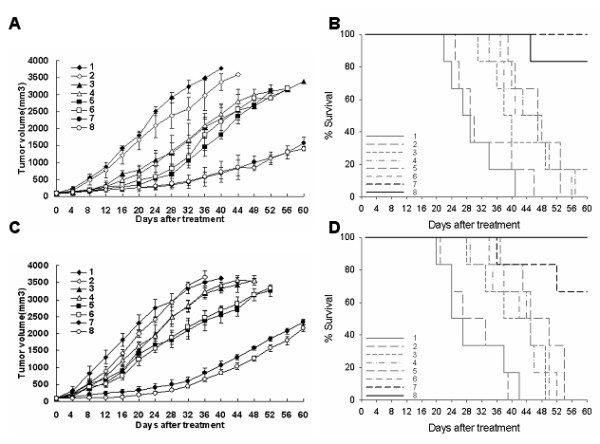
**Ad-hTERT-E1A-Apoptin suppression of melanoma in the C57BL/6 mice model**. (A) Tumor growth kinetics of mice that received intratumorally injections. (B) Survival curve of mice treated intratumorally. (C) Tumor growth kinetics of mice that received intravenously injections. (D) Survival curve of mice treated intravenously. The day that the first injection performed was considered as starting day 0. Data were represented as mean ± SD (A and C). Ad-CMV-E1a-Apoptin or Ad-hTERT-E1a-Apoptin significantly inhibited the growth of tumors in both introtumoral (A) and systemic (C) delivery groups. Although Ad-CMV-Apoptin, Ad-hTERT-Apoptin, Ad-CMV-E1a or Ad-hTERT-E1a had some inhibitory effect on tumors in both experimental groups, the antitumor effects of Ad-CMV-Apoptin and Ad-hTERT-Apoptin in systemic delivery group were marginal (A and C). Furthermore, increased mean survival was also observed in Ad-CMV-E1a-Apoptin or Ad-hTERT-E1a-Apoptin treated mice in comparison with saline, Ad-mock, Ad-CMV-Apoptin, Ad-hTERT-Apoptin, Ad-CMV-E1a or Ad-hTERT-E1a treated mice in the tumor models (B and D). 1. Control; 2. Ad-mock; 3. Ad-CMV-Apoptin; 4. Ad-hTERT-Apoptin; 5. Ad-CMV-E1a; 6. Ad-hTERT-E1a; 7. Ad-CMV-E1a-Apoptin; 8. Ad-hTERT-E1a-Apoptin.

The growth kinetics of the tumors following treatment is shown in Figure 4A. During treatment, compared with control and Ad-mock-infected groups, the tumors in the recombinant adenoviruses groups were suppressed after the first three infections. However, soon after the end of the last injection, the Ad-CMV-Apoptin, Ad-hTERT-Apoptin, Ad-CMV-E1a and Ad-hTERT-E1a infected tumors gradually resumed their growth, whereas most of the Ad-CMV-E1a-Apoptin or Ad-hTERT-E1a-Apoptin infected tumors grew slowly. Intratumoral injection of Ad-CMV-E1a-Apoptin or Ad-hTERT-E1a-Apoptin induced a powerful anti-tumor response. We also evaluated the ability of the recombinant adenoviruses to prolong the survival of the tumor bearing mice (Figure 4B). For ethical reasons, all animals in the study were sacrificed by day 60 before the tumors grew to a volume of 4000 mm^3^. Therefore, the true survival curve of each group of mice was not obtained. However, it is clear from the mean survival results that the mice given Ad-CMV-E1a-Apoptin or Ad-hTERT-E1a-Apoptin showed the longest survival. As shown in Figure 4B, all saline- and Ad-mock-treated animals died between 23 and 46 days after intratumoral injection. After 57 days, all Ad-CMV-E1a-, Ad-hTERT-E1a-, Ad-CMV-Apoptin-, Ad-hTERT-Apoptin-infected mice died, whereas 5/6 and 6/6 of Ad-hTERT-E1a-Apoptin- and Ad-CMV-E1a-Apoptin-infected animals, respectively, were still alive at that point. The mean survival times were 30.5 days for saline-treated mice, 33.7 days for Ad-mock-infected mice, 43.2 days for Ad-CMV-Apoptin-infected mice, 45.8 days for Ad-hTERT-Apoptin-infected mice, 47.8 days for Ad-CMV-E1a-infected mice and 46.5 days for Ad-hTERT-E1a-infected mice. However, tumor bearing mice infected with Ad-CMV-E1a-Apoptin and Ad-hTERT-E1a-Apoptin survived much longer than the other groups and the mean survival was 60 and 57.7 days respectively. The results indicated that Ad-hTERT-E1a-Apoptin conferred significant survival benefits *in vivo*.

### Systemic delivery of Ad-hTERT-E1a-Apoptin suppressed solid melanoma growth

The tumor growth of systemic delivery groups was observed and measured every four days. As shown in Figure 4C, compared with saline-treated and Ad-mock-infected mice, the size impairments of Ad-CMV-Apoptin-treated, Ad-hTERT-Apoptin-treated, Ad-CMV-E1a-treated and Ad-hTERT-E1a-treated tumors were slightly. However, Ad-CMV-E1a-Apoptin and Ad-hTERT-E1a-Apoptin had significant inhibitory effects on the tumors. The tumors infected with Ad-CMV-E1a-Apoptin or Ad-hTERT-E1a-Apoptin started to show growth impairment 12 days and the significant differences among saline-treated, Ad-mock-treated, Ad-CMV-Apoptin-treated, Ad-hTERT-Apoptin-treated, Ad-CMV-E1a-treated or Ad-hTERT-E1a-treated and tumors treated with Ad-CMV-E1a-Apoptin or Ad-hTERT-E1a-Apoptin were confirmed for the entire days follow-up. Consistent with the results of the intratumoral injection experiment, saline-treated and Ad-mock-infected groups had the worst survival, while infection of Ad-hTERT-E1a-Apoptin significantly improved the survival (Figure 4D). The mean survival times were 30.167 days for saline-treated mice, 31.333 days for Ad-mock-infected mice, 41.333 days for Ad-CMV-Apoptin-infected mice, 44 days for Ad-hTERT-Apoptin-infected mice, 48.167 days for Ad-CMV-E1a-infected mice and 43.667 days for Ad-hTERT-E1a-infected mice. However, tumor bearing mice infected with Ad-CMV-E1a-Apoptin and Ad-hTERT-E1a-Apoptin survived much longer than the other groups and the mean survival was 55 and 60 days respectively. This observation demonstrated that intravenous injection of Ad-hTERT-E1a-Apoptin could suppress the syngeneic graft and effectively improved survival.

### Systemic delivery of Ad-hTERT-E1a-Apoptin reduced metastatic lung nodules

**Figure 5:**
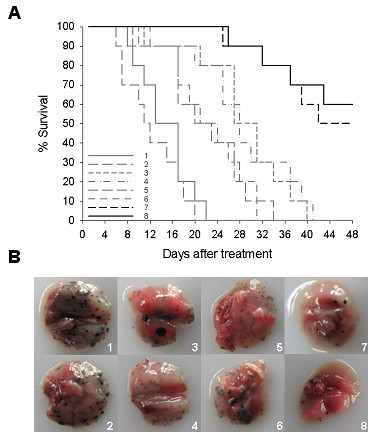
**Ad-hTERT-E1a-Apoptin reduction of pulmonary metastatic melanoma and resulting survival benefits**. (A) Survival curve. (B) Representative photographs of lungs from control and treatment groups. The day that the first injection performed was considered as starting day 0. Saline or Ad-mock treated mice had the worst mean survival and Ad-CMV-E1a-Apoptin or Ad-hTERT-E1a-Apoptin treated mice had the most improved mean survival (A). Furthermore, both numbers and sizes of lung tumor nodules were reduced in mice treated with Ad-CMV-E1a-Apoptin or Ad-hTERT-E1a-Apoptin compared with those treated with saline, Ad-mock, Ad-CMV-Apoptin, Ad-hTERT-Apoptin, Ad-CMV-E1a or Ad-hTERT-E1a (B). 1. Control; 2. Ad-mock; 3. Ad-CMV-Apoptin; 4. Ad-hTERT-Apoptin; 5. Ad-CMV-E1a; 6. Ad-hTERT-E1a; 7. Ad-CMV-E1a-Apoptin; 8. Ad-hTERT-E1a-Apoptin.

Mouse survival analysis showed that Ad-hTERT-E1a-Apoptin or Ad-CMV-E1a-Apoptin treatment significantly increased survival of mice in the lung metastasis model in comparison with the other recombinant adenoviruses-infected or saline-treated mice (Figure 5A). When the experiment was terminated on day 48, 6/10 of Ad-hTERT-E1a-Apoptin- and 5/10 of Ad-CMV-E1a-Apoptin-infected animals were alive, and the median survival time between these two groups did not differ significantly. None of the mice in the other groups were alive at the end of experiment, and the mean survival times were 15.7 days for saline-treated mice, 13.3 days for Ad-mock-infected mice, 29.6 days for Ad-CMV-Apoptin-infected mice, 29.5 days for Ad-hTERT-Apoptin-infected mice, 23.1 days for Ad-CMV-E1a-infected mice and 23.3 days for Ad-hTERT-E1a-infected mice. As shown by the representative metastasic nodules in Figure 5B, Ad-hTERT-E1a-Apoptin significantly decreased tumor burden of the mice. The lungs of mice infected with Ad-hTERT-E1a-Apoptin had minimal metastatic nodules, whereas the lungs from control or treated groups had severe metastasis. Taken together, systemic delivery of Ad-hTERT-E1a-Apoptin significantly reduced tumor burdens and provided survival benefits in a lung metastatic cancer model.

## Discussion

Despite substantial progress in the development of gene therapies and traditional treatments in recent years, the prognosis for many patients with neoplastic diseases remain poor. Cancer gene therapy based on adenoviruses has been extensively studied in pre-clinical and clinical trials. In particular, CRCA has gained increased attention for a number of reasons [6,7]. Because the promoters in these vectors are selective for cancer cells [6,7], these oncolytic viruses have the ability to replicate and to spread to adjacent tumor cells [19]. Furthermore, it has been shown that infection with CRCA generates anti-tumoral immune responses [20] which can complement chemo- and radiotherapies [19]. Importantly, given the proper therapeutic transgenes, CRCA are capable of achieving destruction of primary and distant tumors [7].

In vitro studies showed that a core region of hTERT containing two E boxes and several Sp1 sites is sufficient for the major tumor-selective promoter activity. Many strategies including CRCA have been developed using the hTERT core promoter, containing two E boxes and several Sp1 sites [21], to selectively target tumor cells. One approach being evaluated uses CNHK300, a replicative adenovirus that targets telomerase positive cancer cells [22]. A similar replication-competent adenovirus, AdEHT2, in which hTERT promoter was used to control the expression of the adenoviral E4 gene, was capable of tumor selective replication and oncolysis [23]. Analogous results were also obtained in other replicating adenoviruses, such as Adv-TERTp-E1a [24], hTERT-Ad [25], and Ad/GT-Bax [26], which appear to be promising treatment agents for cancer.

The applicability of cancer therapies is not only determined by their efficiency in eliminating tumor cells; specificity is an equally important prerequisite [3]. Apoptin has such properties which could potentially achieve these objectives. Various research groups have reported that more than 70 analyzed tumor cell lines were proven to be susceptible to Apoptin whereas it does not affecting variety of normal, non-transformed cells such as human endothelial cells, hepatocytes, hematopoietic stem cells, keratinocytes, or smooth muscle cells [27,28]. On the other hand, Apoptin become activated in SV40-transformed normal human fibroblasts or UV-irradiated cells with hereditary cancer-prone syndromes [28,29]. Although, Guelen et al provided data of toxicity of Apoptin towards non-cancerous cells, this study proved cell death only in a fetal cell type and not in other non-transformed cell types [28,30]. However, the safety of apoptin is underlined by the fact that continuous expression of Apoptin under the H2-Kb promoter in transgenic mice does not interfere with lymphocyte development and proliferation [28,31]. While the mechanism by which Apoptin is able to distinguish between tumor and normal cells remains unclear but seems to correlate with its cellular localization. Recently, it was shown that Apoptin-induced apoptosis essentially depends on abnormal phosphatidylinositol 3-kinase (PI3-kinase)/Akt activation, resulting in the activation of the cyclin-dependent kinase CDK2 [32]. Maddika et al indicated that inhibitors of PI3-kinase or Akt not only inhibited CDK2 activation but also protected cells from Apoptin-induced cell death, and Akt-mediated activation of CDK2 was caused by direct phosphorylation as well as by the phosphorylation-induced degradation of its inhibitor p27 (Kip1) [32]. They also identified CDK2 as the principal kinase that phosphorylates apoptin and is crucially required for apoptin-induced cell death [32]. Besides the tumor-selective destruction properties, Apoptin has several important features indicating its application as a novel antitumor agent. One of these characteristics is the ability of Apoptin to induce tumor-specific apoptosis independently of p53 [28,33]. Thus, apoptin is similarly effective in killing tumor cells that are p53-deficient or either express wildtype or mutant p53 [28,33]. Although the role of anti-apoptotic molecules such as bcl-2 in Apoptin-induced apoptosis is still a matter of debate, another important feature of Apoptin is that in certain tumor cell lines it mediated cell death is independent of the Bcl-2 status and is even stimulated by bcl-2 or insensitive to bcr-Abl and bcl-xl [28,29,34]. The main controversy as to the role of bcl-2 may focus on the involvement of Nur77 that can bind to bcl-2 and change its properties from an anti-apoptotic to a proapoptotic molecule [28]. Accordingly, the different expression levels of Nur77 in various cell types might explain the opposite effects of Bcl-2 on Apoptin induced apoptosis. Based on these concepts, it therefore reasonable to anticipate that Apoptin can be used to complement radiotherapeutic and chemotherapeutic approaches.

More than a quarter (27%) of human gene transfer protocols registered with the the Recombinant DNA Advisory Committee (RAC) use adenovirus vectors [35]. The most extensively used first generation human adenovirus (hAd) vectors are replication-incompetent viruses deleted in early region 1 (E1A and E1B) genes. E1 genes that are expressed rapidly upon adenovirus penetration into host cells, are responsible for inducing expression of further viral genes, orchestrating modifications of cellular gene expression and protein activity to favor viral replication [36]. Two genes of this group, E1A and E1B, act in inactivating tumor suppressor Rb and p53 genes that are frequently mutated in cancer cells [7]. Following the generation of E1 deleted hAd vectors, others hAd vectors (e.g., E1 & E3, E2, E4, E2 & E4, or E1, E2 & E4-deleted vectors) were constructed [37]. The products of the E3 gene have been described as nonessential to viral infection and play a role in modulation of the host immune response against virus-infected cells [36]. E2 and E4 genes are involved in multiple processes, such as transcriptional regulation, DNA recombination and virus assembly [36]. Various deleted strategies such as ONYX-015 (dl1520) [38] have been evaluated to be effective in *in vitro *and animal models and led to clinical trials [8,19,39]. Until a fatality case and other reports of inflammation related to adenovirus vector, the use of adenovirus-mediated gene transfer in humans was thought to be fairly benign [35,40]. Because of the short circulatory half life of naked adenoviruses and the neutralizing antibodies existed in most adults, attempts to increase antitumor efficacy through the administration of high doses of adenovirus vectors can lead to liver toxicity and immune response [41-43]. For these reasons, Ad has seen limited clinical use as a systemically administered gene therapy vector. To overcome the potential increased toxicity and reduced vector efficacy during the application of adenovirus vectors, it is important that identifying means to evade innate and pre-existing immunity is a major necessity. Current strategies include use of alternative adenovirus serotypes, modification or chimerism of capsid hexon proteins, generation of hybrid vectors that combine viral and non-viral elements, coating virus with PEG or similar polymers, targeting adenovirus to specific organs, tissues or cell types and so on.

In the present study, we describe the generation of a recombinant adenovirus, Ad-hTERT-E1a-Apoptin, in which replication was driven by hTERT promoter, that selectively replicates and specifically induces apoptosis in tumor cells. When administered to melanoma cells *in vitro*, the anti-tumor effects were evident within 24 h; a single Ad-hTERT-E1a-Apoptin treatment at 100 MOI or 10 MOI completely inhibited the growth of A375 and B16 cells 4 d later, whereas treatment at 1 MOI was less effective. In contrast, the growth inhibition was not observed in HEM cells after treatment with Ad-hTERT-E1a-Apoptin at any MOI. Furthermore, annexin V staining showed that the recombinant adenovirus treated tumors can be divided into two distinct groups: apoptotic and necrotic cells. Although the infection of the recombinant adenoviruses resulted in significant suppression in A375 and B16 cells, the infection with Ad-hTERT-E1a-Apoptin remarkably elevated the percentage of apoptotic cancer cells. These findings indicate that the Ad-hTERT-E1a-Apoptin replicates specifically and induces growth suppression selectively in cancer cells without harming the normal counterparts.

We also observed anti-tumor activity *in vivo *in primary and metastatic tumors, which confirmed and extended the results of the *in vitro *studies. Although the infection of Ad-hTERT-E1a-Apoptin did not lead to complete elimination of the tumors, effective inhibition was observed in both primary and metastatic tumor models. It is plausible that the application of the hTERT promoter allows the adenovirus replication, viral dispersion and transgene expression in any tumor tissues in the animal, regardless of receiving intratumoral injections or not. Additionally, apoptotic tumor cells may trigger dendritic cells to process and present cancer-specific antigens to responding T lymphocytes, resulting in a cytotoxic response and inducing apoptosis in the unaffected tumor cells [44]. Ad-hTERT-E1a and Ad-CMV-E1a also inhibited primary transplantated tumors, but the effects on metastatic tumors were very limited. In the *in vivo *experiments described here, we did not observe any toxic effects after injection of Ad-hTERT-E1a-Apoptin. Thus, our data indicate that there is great potential for improving the safety and efficacy of adenovirus vectors for wide application for treatment of neoplastic diseases.

## Conclusions

Gene therapy with Apoptin offers unique advantages over current approaches for cancer therapy. The fact that Apoptin does not need a functional p53 pathway, is not hindered by the commonly occurring blockage of apoptosis by bcl-2 or bcr-abl, apparently acts downstream of most other factors, and has unparalleled potency suggests that it will be applicable to a wide range of tumors. In addition, the CRCA we engineered induces apoptosis selectively in various cancer cells without adverse effects on normal cells. Furthermore, the *in vivo *and *in vitro *data of this study show that the CRCA expressing Apoptin is more effective but also show that the adenovector dependent on the strong hTERT promoter does not reduce the efficacy of the strategy, thus showing an improvement in the global safety of the CRCA. All these highlights warrant further evaluation for implementation of this strategy in clinical trials.

## Methods

### Cell lines and animals

A375 human malignant melanoma cells, B16 mouse melanoma cells (syngeneic to C57BL/6 mice) and HEK-293 human embryonic kidney cells were obtained from the Committee on Type Culture Collection of Chinese Academy of Sciences (Shanghai, China). A375 and HEK-293 cells were cultured in DMEM. B16 cells were cultured in RPMI 1640. All media (Invitrogen, Beijing, China) above were supplemented with 10% fetal bovine serum (FBS; Hyclone, Beijing, China), 100 units/mL penicillin, and 100 μg/mL streptomycin. HEM human epidermal melanocytes (primary cells isolated from normal human neonatal foreskin) were obtained from ScienCell Research Laboratories (San Diego, CA) and cultured in MelM medium (ScienCell, Carlsbad, CA) supplemented with 0.5% FBS and 100 units/mL penicillin, and 100 μg/mL streptomycin. All cell lines were passaged no more than six months after receipt. DNA-Fingerprinting (monitoring the mutation of human cell lines), isoenzyme analysis (verify the species of origin), Scharfe Casy TT system (evaluating cell proliferation) and Hoechst staining (mycoplasma detection) were used to characterize cell lines by the supplier. Six- to eight-week-old female C57BL/6 mice were purchased from the Experimental Animal Center of the Academy of Military Medical Sciences (Beijing, China) and housed in a pathogen-free facility for all experiments following institutional guidelines.

### Recombinant adenoviruses

Recombinant adenoviruses used in this study were produced with the Adeno-X Expression System (BD Biosciences Clontech) following the manufacturer's instructions. Briefly, the transgene cassettes containing the hTERT core promoter (the 5' flanking region of the hTERT gene between positions -283 to -78) driving E1a and the CMV promoter driving Apoptin were subcloned into the BD Adeno-X Viral DNA (the adenoviral genome) via the shuttle vector pShuttle2. Then the infectious adenovirus designated as Ad-hTERT-E1a-Apoptin was packaged in HEK-293 cells. Similar strategies were used to generate the other recombinant adenoviruses designated as Ad-CMV-Apoptin, Ad-hTERT-Apoptin, Ad-CMV-E1a, Ad-hTERT-E1a and Ad-CMV-E1a-Apoptin, and the Ad-Mock was generated using the adenovirus backbone only (Figure 1A). The purification and titration of the amplified virus were performed using Adeno-X Virus Purification kit (BD Bioscience Clontech) and Adeno-X Rapid titer kit (BD Bioscience Clontech), respectively.

### Western blot analysis

Cells were infected with the various adenoviruses at a multiplicity of infection (MOI) of 100 for 48 h. The expressions of E1a and Apoptin were analyzed by Western blot as described previously [45]. The primary antibodies were anti-E1a (1:10,000; mouse monoclonal; Abcam, Cambridge, MA) for E1a detection and anti-Apoptin (1:1,500; rabbit polyclonal, a kind gift from Mi Zhiqiang, Jilin University, China) for Apoptin detection, and the secondary antibodies were horseradish peroxidase(HRP)'conjugated anti-mouse and anti-rabbit IgG (1:2,500; Abcam), respectively. The bands were visualized with Pierce ECL Western Blotting Substrate (Pierce, Shanghai, China). Extracts of Ad-mock-infected cells was used as negative control and detection of GAPDH was used as an internal control.

### Cell viability assay

Cell viability was determined by standard 3-(4,5-dimethylthiazol-2-yl)-2,5-diphenyl tetrazolium bromide (MTT; Sigma, St. Louis, MO) assays as described previously [45]. Cells were infected with various concentrations (1 MOI, 10 MOI, and 100 MOI) of the recombinant adenoviruses, and the cell viability was then measured every day over a 4 d period. The percent cell death was expressed with respect to control values using the following formula: [100 × (control cells -experimental cells)/(control cells)] [46,47].

### Flow cytometry analysis

Cells were infected with the recombinant adenoviruses (100 MOI) for 48 h, trypsinized and washed once with PBS. The cells (1 × 10^6^) were resuspend in binding buffer and stained with FITC-labeled Annexin V (Annexin V-FITC Apoptosis Detection Kit; BioVision, Mountain View, California) according to the manufacturer's protocols. To exclude late apoptotic and necrotic cells, propidium iodide (PI) was added to the FITC-Annexin V-stained samples. Then, the samples were examined by flow cytometry (FACScan, Becton Dickinson, Franklin Lakes, NJ) for apoptosis analysis.

### Fluorescence assay

Fluorescent immunostaining was performed on cells double-stained with FITC-labeled Annexin V and PI following the Annexin V-FITC Apoptosis Detection Kit (BioVision) manufacturer's instructions. The cells were then observed under a fluorescence microscope (BX51; Olympus) and the images of representative cells were captured with a CCD camera (DP71; Olympus). Samples were processed simultaneously, and all images were captured with the same parameters.

### Animal experiments

The *in vivo *anti-tumor experiments were performed in three independent models. In the first two models, 1 × 10^6 ^B16 cells were implanted subcutaneously into the right flank of C57BL/6 mice. Once the tumor size reached 50 to 100 mm^3^, mice were randomly assigned to 8 treatment groups (6 mice per group). After establishment of the tumors, the mice of the first model received intratumoral injections of various recombinant adenoviruses at a dose of 1 × 10^9 ^plaque-forming units (pfu) in 50 μl of saline, and the control group received 50 μl of saline alone. In the second model, the injections were administered via the tail vein. All injections were given every two days for the first week (day 6, 8 and 10 after implantation) and once weekly for two more weeks (day 17 and 21 after implantation). Tumor size was measured using calipers every four days and calculated with a formula of [0.52(smallest diameter )^2^(largest diameter )] [45,48]. In the third model, 1 × 10^6 ^B16 cells were administered via tail vein. Six days after implantation, the mice were treated according to the injection protocol of the second model. During the tumor study, all animals were monitored daily and sacrificed at the end of the experiment.

### Statistical analysis

The statistical significance of differences was done using one-way ANOVA and statistical significance was accepted as *P *< 0.05. Log rank tests were used for survival analysis. Data from all animals are represented in the Kaplan-Meier plots.

## Competing interests

The authors declare that they have no competing interests.

## Authors' contributions

LX and JNY designed the study and wrote the manuscript. LX, LY, WZM and LC performed virus construction, LHJ, TMY, JKS and SLL performed molecular studies. LX, GP, YEC and XXH performed cell studies. LX, KSF, WZY and WYH performed animal studies. LX and JNY performed statistical analysis and data interpretation. All authors read and approved the final manuscript.
